# Mind the mouth: Are doctors doing inadvertent harm by not learning about dentistry and oral health?

**DOI:** 10.1177/10398562261431180

**Published:** 2026-03-11

**Authors:** Ava Elizabeth Carter, Alisha Johnson

**Affiliations:** Psychiatry Department, Deakin Private Hospital, Deakin, ACT, Australia; Medical School, The Canberra Hospital, 2219 Australian National University, Canberra, ACT, Australia; Australian Centre for Integration of Oral Health (ACIOH), School of Nursing & Midwifery, Western Sydney University, Ingham Institute Applied Medical Research, Sydney, NSW, Australia

**Keywords:** oral health, psychiatry, mental illness, dentistry, medical education

## Abstract

**Background:** Oral and mental health are closely linked, yet Australia’s healthcare system continues to treat them in silos – particularly to the detriment of its most vulnerable populations.

**Analysis:** People living with mental illness face a significantly increased risk of oral diseases, including dental caries, periodontitis, and oral lesions, which concurrently worsen their mental health conditions. Despite strong evidence of this bidirectional relationship, oral health remains largely absent from mental health policy, psychiatric care pathways, and medical education frameworks. Equally, mental health remains largely absent from dental education frameworks. Current clinical training lacks sufficient integration across disciplines, limiting the capacity of clinicians to provide holistic, patient-centred care.

**Conclusions:** To reduce health inequities and improve long-term outcomes, coordinated policy action is needed to embed oral health in mental healthcare delivery, promote interdisciplinary education, and prioritise oral health as a key component of the social determinants of health in Australia.

## Introduction

People with severe mental illness (SMI) experience poorer oral health compared to the general population with this population experiencing higher rates of advanced dental disease and oral health needs identified in key systematic reviews.^1,2^ It is well established in the literature that poor oral health alone can have a negative impact on an individual’s physical health, contributing to the development of diabetes,^3^ cardiovascular diseases,^4^ and dementia.^5^ Poor oral health, combined with the increased risk of metabolic-related illness secondary to psychotropic medication side effects as well as impact on oral health from mental illness symptomology, is a triad for poor overall health and an unfortunate reality that this highly vulnerable group faces.

## Background

Mental health and oral health have evolved as separate domains of healthcare, leading to significant missed opportunities for integrated holistic care.

Education and professional training have been previously identified as major enablers.^6^ Despite this, oral health is seldom emphasised in medical training or practice^7^ despite oral health included in clinical guidelines.^8^ Psychiatrists are taught little to nothing about oral health, and there are no integrated clinics that allow simple, quick, and efficient referrals in Australia that are co-located within psychiatric units or clinics. Even though some public health dental clinics are located in the same vicinity as some gazetted mental health units, there are additional barriers faced in facilitating attendance at a clinic appointment including transportation, staffing for accompanied leave, and risk associated with absconding or harm.

As healthcare organisations, lived experience groups, and governments push for increasing multidisciplinary collaboration,^9^ the environment for integrated care is at a turning point and presents an opportunity for igniting the integration of oral and mental health through policy reform, integrated models of care, and foundation education in medical and dental schools.

## Why investing in oral health mental health integration makes sense

The first dental school, the Baltimore College of Dental Surgery, was founded in 1840 and marked the formalisation of dentistry as a separate profession to medicine.^10^ As licensing bodies and professional associations such as the American Dental Association began to structure dentistry under different codes of conduct and professional standards, dental practice became a profession distinct from general medicine, operating largely outside of hospital systems, which has made it unique in its lack of integration into most hospital on-call rosters, and lacking in any formal internship or residency program that medical doctors train within. The teaching of oral health is thus segregated and taught almost entirely separate to medicine. Whilst some may argue that programs such as physiotherapy or speech therapy are similar, the key differences lie in the allied health disciplines integration into hospital intern teaching sessions, their inclusion in medical school programs, standardised on call availability in hospitals, and liaison services in community settings which are publicly funded.

Those living with SMI form a population group which is at particular risk of poor oral health and its subsequent systemic complications.^11–13^ Oral health is rarely included in routine assessments within clinical psychiatric settings, despite clear evidence of its correlation on mental and physical health, where individuals with SMI present with significant higher rates of untreated dental caries, periodontal disease, and edentulism.^1,14,15^ Whilst there is a clear correlation of increased rates of poor oral health, antipsychotic and tricyclic medications often worsen the situation, as they cause xerostomia, increasing the risk of dental decay and gum disease,^16^ and substance use further compounds these effects.^9,17^ Furthermore, poor oral health contributes to pain, chronic inflammation, and systemic illness, all of which can exacerbate psychiatric symptoms of psychiatric illness, but also of other chronic diseases.^18^ Chronic gum disease is increasingly linked to mental health disorders and systemic conditions through elevated inflammatory markers like IL-6 and CRP, which can disrupt brain function by affecting neurotransmitter metabolism, neuroplasticity, and stress-response systems.^19,20^ Emerging evidence also connects periodontal disease to neurodegenerative conditions such as Alzheimer’s, highlighting the importance of incorporating oral health into mental healthcare.^20^

If physical illness was not a substantial enough reason to invest in the integration of oral and medical services, quality of life and the potential years of productivity lost to disability and the economic costs associated should be. In 2024, poor oral health resulted in a burden of 131,935 disability-adjusted life years (DALYs) in Australia. This represents a cost of 4.5 DALYs per 1000 persons.^9,17,21^ In 2021–22, Australia spent $11.1 billion on dental services, more than half of which is paid for by the individual.^21^ There were also approximately 83,000 hospitalisations for preventable dental conditions in 2020–21. When estimating conservatively, a hospital bed costs $1800 per day, which is $150 million dollars of unnecessary expenditure.^21^

Public policy also has a critical role in shaping population oral health, and failure to act carries significant costs for both health systems and broader economic productivity. The Australian Bureau of Statistics reports that over one in three Australians aged 15 and over avoided or delayed visiting a dentist in 2021 due to cost, with rates disproportionately higher among low-income and regional populations.^17^ Despite oral disorders accounting for 2.3% of the total disease burden and consuming 4.8% of overall health expenditure, only 1.3% of national health spending is directed toward dental services. Recent data show that dental care receives just 1.2% of the Commonwealth’s $105 billion health budget and 1.5% of state and territory health allocations totalling $65 billion.^9^ This mismatch between disease burden and investment reflects a systemic misalignment that perpetuates inequities and drives up long-term costs through preventable complications and hospitalisations.

Patients with SMI also routinely experience more pain, difficulty speaking, anxiety, embarrassment, and challenges with eating than those who do not suffer SMI.^9,22,23^ As gum disease prevalence continues to rise nationally from 23% in 2004–06 to 30% in 2017–18, the cost to the individual, public services, and the nation will continue to rise.^9,17^

Despite the evidence, and oral health being stipulated in clinical guidelines as a consideration in care planning,^8^ few psychiatrists receive training in oral health assessments or referral pathways – a missed opportunity, particularly given that individuals with severe mental illness are twice as likely to experience total tooth loss and less likely to access regular dental care.^16^ Integrated co-located services would strengthen interprofessional collaboration between oral and mental health services by reducing barriers for referrals, whilst improving patients’ access to care.

## Towards integrated education

A survey of Australian medical students found that few institutions offer formal oral health education such as at Griffith University.^24^ As a result, doctors are not trained to recognise dental emergencies, understand the psychological impacts of oral infections, or appreciate the oral biome’s role in holistic health. Similarly, dental students are educated in isolation from other health professions, which is a key problem and perpetuates the idea that oral health is separate from mental and physical health. A study of 65 dental schools in the United States and Canada found that only seven offered joint didactic teaching with other health disciplines, and just two facilitated interprofessional projects.^25^

Promising models are emerging however, and Australia can build bridges and close this widening diastema of inequality for oral healthcare. In 2022, Morel and colleagues piloted an oral health clerkship for U.S. medical students using the Smiles for Life curriculum.^26^ Third-year students who participated demonstrated significant improvements in oral health knowledge and confidence in performing oral examinations.

In other caring professions, the integration of oral health into nursing and midwifery undergraduate programs has shown significant improvements in nursing students’ oral health attitudes and confidence.^27^ Similarly, the potential of such training in the foundational years of medical school could ensure that future physicians can identify oral health problems, collaborate with dental professionals, and deliver holistic care to patients with complex co-morbidities. This training would not only assist future psychiatrists but also other medical specialities when considering individuals with a mental illness are at an increased risk of other medical co-morbidities. It may also provide opportunity for the identification of oral diseases through their contact with other specialities.

This integration of education into medical programs supports the Australia’s National Oral Health Plan 2015–2024 which advocates for greater integration of oral health into general health services and education.^28^ Achieving this will require collaboration between medical and dental faculties to develop shared learning objectives, clinical placements, and interprofessional education. Core competencies should include oral examination techniques, recognition of oral manifestations of systemic disease, and understanding the oral side effects of psychiatric medications.

## Conclusion

Oral health is fundamentally intertwined with mental health, yet it remains overlooked in clinical practice, medical education, primary care, and health policy. Addressing this systemic neglect demands coordinated action across multiple levels: embedding oral health within psychiatric assessment and treatment, incorporating oral health training into medical curricula, prioritising culturally appropriate care for Indigenous communities, and implementing policy reforms that guarantee equitable access to dental services. The long-term benefits to both population health and government expenditure through reduced preventable hospitalisations and improved overall wellbeing are well documented. As Australia aspires to a truly integrated and equitable healthcare system, oral health can no longer be treated as peripheral. It must be firmly positioned as a foundational element of comprehensive medical and psychiatric care.

## Data Availability

There were no data sets used in this manuscript.*
